# DNA Methylation and Cancer Diagnosis

**DOI:** 10.3390/ijms140715029

**Published:** 2013-07-18

**Authors:** Yannick Delpu, Pierre Cordelier, William C. Cho, Jérôme Torrisani

**Affiliations:** 1 Cancer Research Center of Toulouse Inserm UMR 1037, 31432 Toulouse cedex 4, France; E-Mails: yannick.delpu@inserm.fr (Y.D.); pierre.cordelier@inserm.fr (P.C.); 2 University de Toulouse III-Paul Sabatier, 118 Route de Narbonne, 31062 Toulouse cedex 9, France; 3 Department of Clinical Oncology, Queen Elizabeth Hospital, Hong Kong, China; E-Mail: williamcscho@gmail.com

**Keywords:** DNA methylation, biomarkers, cancer diagnosis, DNA methylation inhibitors, clinical trials

## Abstract

DNA methylation is a major epigenetic modification that is strongly involved in the physiological control of genome expression. DNA methylation patterns are largely modified in cancer cells and can therefore be used to distinguish cancer cells from normal tissues. This review describes the main technologies available for the detection and the discovery of aberrantly methylated DNA patterns. It also presents the different sources of biological samples suitable for DNA methylation studies. We discuss the interest and perspectives on the use of DNA methylation measurements for cancer diagnosis through examples of methylated genes commonly documented in the literature. The discussion leads to our consideration for why DNA methylation is not commonly used in clinical practice through an examination of the main requirements that constitute a reliable biomarker. Finally, we describe the main DNA methylation inhibitors currently used in clinical trials and those that exhibit promising results.

## 1. Introduction

### 1.1. DNA Methylation, a Physiological Process

Developmental processes and proper biological functions are tightly dependent on hierarchical and regulated gene expression patterns. Numerous molecular processes control gene expression. DNA methylation is a physiological epigenetic process that leads to long term-repression of gene expression [1]. Like histone modifications, DNA methylation does not impact genomic DNA sequence itself, but adds a methyl (CH_3_) group on cytosines of CG dinucleotides. This reaction is catalyzed by a DNA methyltransferase enzyme family composed of DNMT1, DNMT3a and DNMT3b [1]. Briefly, this chemical modification affects gene expression through two major mechanisms. DNA methylation can directly interfere with the binding of transcription factors that are sensitive to methylated CpG islands (p for phosphate) [1]. Alternatively, methylated cytosines are recognized by the methyl binding domain (MBD) protein family that mediates the recruitment of chromatin remodeling enzymes such as histone deacetylases [1]. Consequently, histone deacetylation leads to the condensation of the chromatin and to the silencing of the neighboring gene [1]. Even though present all along the genome, CG dinucleotides are not equally interspaced and are frequently grouped in CpG islands [1]. DNA regions targeted by DNA methylation are physiologically subject to long term silencing. They usually correspond to genes transiently expressed during development, subject to X chromosome inactivation, imprinted genes or vestigial repeated sequences [2]. Approximately sixty percent of our genes harbor one or more CpG islands in their promoter and therefore can be potentially silenced by DNA methylation. Meanwhile, in normal cells only 5% of these promoters are methylated illustrating that the establishment of this epigenetic mark is not a predominant process. However, CpG islands are not exclusively located at gene promoters, many of them are located within the gene body. Conversely, methylation of intragenic CG dinucleotides can increase transcription and/or activate an alternative promoter [1,3].

### 1.2. DNA Methyltransferase Family and Establishment of DNA Methylation Profiles

The first identified DNA methyltransferase DNMT1 is known as a maintenance DNMT [4]. It is responsible for the exact copying of the DNA methylation pattern on the neo-synthesized strand during DNA replication. Therefore it principally localizes to the DNA replication fork. Due to its importance in DNA replication, DNMT1 expression is tightly regulated during the cell cycle by several mechanisms and maximal expression occurs during S phase [4]. Since its role is to ensure the inheritance of the DNA methylation pattern through cell division, DNMT1 expression is maintained after development. From a transcriptional point of view, two transcript variants of DNMT1 mRNA were identified: a full-length form of 1,616 amino acids, and an oocyte-specific variant that lacks the N-terminal 118 amino acids of the full-length form (DNMT1o) but both are enzymatically active [4]. DNMT1 is capable of *de novo* methylation but its affinity for unmethylated DNA is far lower than for hemi-methylated DNA. As an illustration of the crucial role of DNMT1, the genetic loss of *DNMT1* gene in the mouse model is embryonic lethal [5].

The *de novo* DNA methyltransferases DNMT3a and DNMT3b are responsible for the establishment of DNA methylation patterns during development. They are highly expressed during embryogenesis [4]. Similarly to DNMT1, DNMT3a and 3b expression is increased in S phase but they do not localize at the DNA replication fork [5,6]. Immuno-fluorescence studies show that both *de novo* DNMTs localize to heterochromatin 6, and further experiments demonstrate that DNMT3a and DNMT3b are strongly associated to nucleosomes containing methylated DNA, and promote propagation of DNA methylation through stabilization of those enzymes [7,8]. The *DNMT3a* gene encodes at least two protein products, both enzymatically active but with variation on their localization in the nucleus. The *DNMT3b* gene encodes five isoforms: two are active and three inactive [4]. Conversely to DNMT1, as development progresses both genes undergo tissue-specific repression such that their expression is scarcely detectable in adult tissues [9]. De *novo* methylation is a crucial developmental process as the *DNMT3b* knockout is lethal at the embryonic stage of mouse development [9,10]. DNMT3a-deficient mice are viable only 4 weeks after birth [9]. An additional DNMT3-like enzyme (DNMT3L) was identified. It is highly similar to DNMT3a and 3b, but lacks the catalytic domain [11]. Interestingly, DNMT3L is expressed simultaneously with DNMT3a and DNMT3b, and despite its absence of enzymatic activity, it stimulates *de novo* methylation *via* its interaction with these enzymes [11].

A further enzyme associated with the DNMT family based on sequence homology is named DNMT2, though it shows no DNA methyltransferase activity. Homozygous deletion of the DNMT2 gene in mouse ES cells has no effect on the maintenance or the establishment of methylation, providing evidence that DNMT2 does not play a major role in global *de novo* or maintenance methylation of CG sites in mammals [12]. Other studies demonstrate that DNMT2 methylates transfer RNAs [13–15]. Consequently, DNMT2 is now known as TRDMT1 (tRNA aspartic acid methyltransferase 1) by the HUGO gene nomenclature.

### 1.3. DNA Methylation Alterations in Cancers and Preneoplastic Lesions

Alteration of DNA methylation patterns is a hallmark of cancer [16]. Numerous studies describe repression of tumor suppressor genes (TSG) involved in various cellular pathways (cell cycle, apoptosis or genome maintenance) during carcinogenesis by DNA hypermethylation of their promoters. Paradoxically, cancer cells exhibit a global genome hypomethylation that leads to genomic instability and re-expression of silenced genes [16,17]. Mechanisms underlying this paradox are still not clearly explained. Wild and Flanagan depict current knowledge on genome wide DNA hypomethylation associated with cancer [18]. Briefly, two competing theories of “passive” *vs.* “active” demethylation processes could explain this phenomenon. The former implies a disruption of the link between histone modifications and DNA methylation establishment, an aberrant localization of DNMT1 to DNA damage sites or a metabolic imbalance favoring a decrease in the methyl group donor, *S*-adenosyl-methionine. Conversely, the latter theory relies on a class of enzymes harboring a demethylase activity. The TET protein family (Ten Eleven Translocation proteins) is described to actively demethylate methyl-cytosines by their oxidization and elimination through different mechanisms in physiological conditions [19]. Briefly, the TET enzyme family facilitates passive DNA demethylation by oxidizing methyl-cytosines to 5-hydroxyl-methylcytosines (5 hmC) leading to a considerable reductions in UHRF1 binding (ubiquitin-like containing PHD and RING finger domains) and in DNMT1 methyltransferase activity at the replication fork [20,21]. A second mechanism involves the DNA repair pathway. Hydroxy-methylcytosines are converted either by further oxidization or by deamination that leads to a nucleotide mismatch, which will be excised and replaced by a cytosine [22,23]. Last, DNMT3a demonstrates methyltransferase activity in reducing conditions and conversely, dehydroxymethylation in oxidizing conditions that converts 5 hmC in cytosines [23].

Recent studies report that the induction of TET suppresses breast tumor growth, invasion and metastasis in mouse xenografts [24,25]. Moreover, TET down-regulation in hepatocellular carcinoma correlates with a decreased level of 5 hmC and is associated with tumor size and poor overall survival [26]. Taken together, these observations are controversial, with a pro-oncogenic effect of TET mediated-DNA demethylation.

AID proteins (apolipoprotein B mRNA editing catalytic polypeptides) mediate deamination of cytosines to uracils. This chemical reactions leads to mutations in mRNAs that are essential for the generation of the vast repertoire of antibodies in mammals [27]. AID proteins are also shown to play a role in active DNA demethylation, as down-regulation of AID in heterokaryons blocks the rapid demethylation normally observed at the OCT4 and NANOG promoters [28]. This strongly suggests that DNA demethylation mediated by AID is not a global effect but rather AID can target specific loci through unknown mechanisms. Moreover, remarkable studies from Métivier and co-workers highlight a demethylase activity for DNMT3a and -3b in association with DNA glycosylase and base excision repair machinery. This demethylase activity is involved in cyclical methylation and transcription of the pS2 gene promoter [29]. Once again, this demethylation activity seems cyclical and does not explain the global and long term demethylation observed in cancer. Hence, in the absence of indisputable identification of the demethylation mechanisms leading to the global hypomethylation associated with cancer, current knowledge may favor slow and passive demethylation during carcinogenesis. Given the global rearrangement of the methylome, it is unlikely that all methylation changes play a causative role in carcinogenesis. Kalari and Pfeifer elegantly introduce the concept of “driver” and “passenger” DNA methylation alterations in cancer. Indeed, DNA hypermethylation of TSG promoters can be easily associated with a carcinogenic effect, and so be referred to as a “driver” alteration. Conversely, a particular chromatin environment predisposing a gene to DNA hypermethylation with no particular effect on cell transformation can reflect a “passenger” event [30].

If aberrant DNA hypermethylation has been described in cancers for a long time, such alterations in pre-cancerous lesions are now documented. This constitutes an interesting field of investigation to fully understand key events of carcinogenesis. DNMT1 over-expression in pre-cancerous lesions of the pancreas has been reported [31] and early DNMT over-expression is also observed in several rodent models [32,33]. Despite moderate DNMT over-expression in pre-neoplastic lesions, many studies measure changes in DNA methylation in early steps of various cancers. They constitute indirect proof that DNMT mis-regulation is an early event of carcinogenesis. Sato *et al.* reports that pancreatic cancer precursor lesions display aberrant DNA hypermethylation at early stages and the prevalence increases progressively during neoplastic progression [34]. Similarly, we describe that the DNA region encoding the miR-148a is hypermethylated in the early stages of pancreatic cancer [35]. DNA hypermethylation of *hMLH1* and *MGMT* is found in another type of pancreatic pre-cancerous lesions [36]. Alteration in DNA methylation increases from normal gastric mucosa to pre-neoplastic lesions and then cancerous lesions of the stomach [37]. *GSTP1* promoter hypermethylation is detectable as early as prostatic intraepithelial neoplasia [38].

### 1.4. Altered Expression of DNMTs in Cancers

Despite no evidence of clearly identified actors in DNA demethylation, alteration of global DNA methylation patterns in cancer is often associated with an over-expression of DNMTs as described in various tumors such as pancreas, colon, breast, and acute and chronic leukemia [39–42]. The mechanism by which DNMT over-expression leads to aberrant DNA methylation patterns remains unclear. Robertson *et al.* demonstrates that the exact degree of over-expression of DNMTs in tumors remains controversial but a low-level over-expression seems to be common [43]. In addition, the mutation of *TET2* in acute myeloid leukemia (AML) is associated with a decrease in 5 hmC content and, by the impairment of the demethylase pathway. This mutation could play a role in the DNA hypermethylation observed in cancer [44]. The mechanisms that explain DNMT over-expression are various. Esteller *et al.* observes the duplication of the *DNMT3b* gene in different cancer cell lines where copy number correlates to increased mRNA and protein levels [45]. Additionally, the same group showed that *DNMT3b* over-expression occurs through the stabilization of its mRNA in human colorectal carcinoma RKO cells [46]. In addition to an increased amount of DNMTs, several mechanisms are proposed to be involved in the methylome rearrangement during carcinogenesis. Inappropriate timing in the expression of *DNMT*s during the cell cycle could lead to the establishment of methylation marks, and further, their aberrant localization may target uncommon DNA sequences or lead to abnormal protein-protein interaction [43].

AML is known to be associated with frequent *DNMT3a* mutations. Measurements of mutation frequency of the *DNMT3a* gene in AML reveal that 22% of AML patients display mutations predicting translational consequences. These mutations mainly occur at the amino acid R882. Interestingly, gene expression analysis does not show specific expression profiles in cells expressing *DNMT3a* mutants. To our knowledge, the exact functional consequences of such changes are not detailed but are associated with a shorter median overall survival (12.3 months *vs.* 41.1 months) [47]. Moreover, no mutation is found in *DNMT1*, *DNMT3b* and *DNMT3L* genes, suggesting a relative specificity of the *DNMT3a* mutation for AML.

## 2. DNA Methylation Studies in Biological Samples

The discovery of alterations in DNA methylation in cancer cells is offering scientists an alternative field of investigation to differentiate tumor cells from normal cells. This is complementary to the genetic and cytological analyses used to date to improve cancer diagnosis. A large panel of molecular approaches has been developed to study DNA methylation profiles from a variety of biological samples. They differ by the number of DNA regions studied, their sensitivity, reproducibility, resolution, duration, and cost. Here, we describe some of the most appropriate techniques that can be used for cancer diagnosis. We also describe the next generation technologies that will allow for the identification of new methylated DNA markers to further improve cancer diagnosis.

### 2.1. Most Common Approaches for DNA Methylation Studies

Almost forty years have passed since scientists were able to quantify 5-methylcytosine content in genomic DNA by high-performance liquid chromatography (HPLC) [48], high-performance capillary electrophoresis (HPCE) [49] or nearest-neighbor analysis [50]. Although these approaches provide accurate percentages of methylated cytosines, they lack information on the location of methylated cytosines on the genome. Whereas global DNA hypomethylation is proposed as an useful biomarker for liver cancer diagnosis [51], it seems difficult to implement these techniques for cancer diagnosis in clinical usage. The exact positioning of methylated cytosines became possible with the use of methylation-sensitivity restriction enzymes (MSRE) combined with polymerase chain reaction (PCR). This method requires an extremely small amount of starting DNA material; however, the resolution of methylation patterns is limited to the number of restriction sites in a given DNA region. Genome-wide implemented methods are proposed such as Restriction Landmark Genome Scanning (RLGS) [52], amplification of inter-methylated sites (AIMS) [53] or methylation arbitrarily primed PCR (Ms-AC-PCR) [54] but hardly applicable for cancer diagnosis in the clinical routine.

Sodium bisulfite treatment of genomic DNA revolutionized DNA methylation studies [55]. It consists in a chemical reaction that modifies unmethylated cytosines into uracils and does not affect methylated cytosines. Methylation patterns for specific DNA regions or the entire genome is obtained from bisulfited DNA using PCR-based or microarray approaches. Modified DNA is amplified by PCR using dedicated PCR primers that confer sequence specificity and a high sensitivity. DNA bisulfite conversion has been used for the development of a large panel of different molecular approaches. As one of them, bisulfite mapping allows the determination of DNA methylation patterns for specific DNA regions spanning approximately 100 bp to 1 kb. Several sequencings for a given PCR product are required to obtain precise and quantitative description of DNA methylation patterns. This classical technique is inexpensive, reproducible and with high resolution. Even though this approach is poorly sensitive and time consuming, it is frequently used to finely map the precise methylation level of nucleotides. Methylation specific-PCR (MS-PCR) is a method that derives from the latter one in which two sets of PCR primers are specifically designed to amplify methylated and unmethylated DNA regions of interest [56]. The detection of PCR products is originally performed by gel electrophoresis. This technique has been replaced by Quantitative MS-PCR (qMS-PCR), in which PCR amplification is monitored in real time by the incorporation of fluorescent molecules. This improvement allows for precise quantification of the DNA methylation levels of numerous specific regions and avoids the long electrophoresis step. Quantitative multiplex MS-PCR (QM-MS-PCR) and one step MS-PCR (OS-MS-PCR) [57] are also available to co-amplify specific genes in tissues from different origins or to determine DNA methylation levels of a specific region without the DNA extraction procedure. Since they only provide the methylation status of few CpG sites (contained in PCR primers), qMS-PCR requires perfect knowledge of the most discriminative methylated regions present in cancer cells to design powerful primers for diagnosis. As these approaches provide quantitative measurements of DNA methylation, it is necessary to define a cut-off DNA methylation value before declaring that a sample is positive [56]. Nevertheless, qMS-PCR techniques are simple, rapid, inexpensive, highly-sensitive and easily standardized. They are currently one of the most commonly used techniques for cancer diagnosis in clinical use. Methylation-sensitive high-resolution melting (MS-HRM) is based on the fact that the nucleotide sequence of PCR products of bisulfite-treated DNA will differ depending on the methylation status of the DNA region of interest. The methylation level is determined by comparing the melting dissociation curves to standard PCR products of the same region containing known methylated CpG sites. Despite its high sensitivity, this method requires the acquisition of specific PCR apparatus and skilled operators. COBRA, for combined bisulfite restriction analysis, uses the ability of bisulfite conversion to create new restriction enzyme sites or to maintain consensus sites of MSRE. After amplification, PCR products are digested with appropriate MSRE. The proportion of digested PCR products is compared to undigested PCR products by poly-acrylamide gel electrophoresis and image quantification software. This technique is reliably applied to DNA obtained from formalin-fixed paraffin embedded (FFPE) tissue sample [58]. Moreover, this approach allows for the assessment of the DNA methylation of a large number of biological samples. This technique is, however, limited by the presence of restriction sites in the sequence of interest after bisulfite treatment. More recently, high throughput approaches have been developed. For instance, Methyl Light is a high throughput quantitative methylation assay that uses fluorescent-based real time PCR (TaqMan^®^, Applied Biosystems, Forster City, CA, USA) in combination to bisulfite treatment [59]. Also combined with bisulfite treatment pyro-sequencing is a quantitative DNA sequencing method in which light is emitted as a result of an enzymatic reaction representing each time a nucleotide is incorporated into the growing DNA chain [60]. The approaches are limited by a short length of studied DNA and require exclusive apparatus. However, both these quantitative techniques detect low amounts of methylated DNA in heterogeneous DNA preparation. Easily standardized, rapid and inexpensive, these techniques are increasingly used for clinical purpose.

Beside the different approaches used for cancer diagnosis, several genome-wide technologies have recently been developed. Less applicable for clinical practice, these next-generation technologies are perfectly adapted for the discovery of new DNA methylation biomarkers. Classical chromatin immuno-precipitation followed by array hybridization (ChIP on chip) identified numerous aberrantly methylated genes in cancers. Basically, methylated DNA is purified using MBD protein domains or antibodies directed against 5-methylcytosine (methyl-DIP) and subsequently hybridized on genomic microarray [61]. Although these approaches allow for large scanning of the genome, they do not provide direct proof of DNA methylation. Validation analysis is often recommended to prove actual difference in methylation levels. More recently, next-generation sequencing (NGS) technologies significantly increased the resolution level of DNA methylation profiles. For instance, ultra-deep sequencing using 454 sequencing apparatus (Roche) provides the methylation of 25 genes in more than 40 samples in a reduced amount of time [62]. NGS can also be adapted to immuno-precipitated DNA fragment (Methyl DNA Immuno-precipitation sequencing also called MeDIP seq). Ultimately NGS permits the sequencing of the entire genome after bisulfite conversion. Although this new approach requires bio-informatic expertise, whole genome bisulfite sequencing (WGBS) recently revealed new differentially DNA methylated regions in chronic lymphocytic leukemia cells [63]. Beside these NGS approaches, high throughput single nucleotide polymorphism (SNP) genotyping systems are suitable for DNA methylation analysis from bisulfite-converted genomic DNA [64]. For instance, the methylation analysis of 1,500 CpG from 371 genes in 96 samples (Golden Gate^®^ BeadArray-Illumina) allowed the identification of a panel of adenocarcinoma-specific methylation markers. This technology was implemented and now allows for the methylation analysis of approximately 450,000 CG sites spread along 99% of human genes (Infinium Human Methylation 450-Illumina). Using this approach, Fuks’ group highlighted the existence of previously unrecognized breast cancer groups, therefore improving diagnosis of this cancer [65].

### 2.2. Variety of Biological Samples for DNA Methylation Studies

Owing to its capacity to resist extreme conditions, DNA can be obtained from a large panel of tissue samples or biological fluids. Moreover, highly sensitive techniques (see above) can detect aberrantly methylated DNA from small amounts of DNA or diluted within DNA from normal cells. Biological samples differ by their accessibility and enrichment in tumor cells and organ specificity. After surgery, solid tumor (primary tumors or metastasis) samples constitute the samples of choice for DNA methylation studies. They are enriched in tumor cells and provide significant amounts of DNA. Archived samples with corresponding clinical annotations such as FFPE tumor sections are also used to detect methylated DNA; these samples are restricted to patients who are subject to surgery and DNA from solid tumors is obtained after biopsies or biopsy washing [35]. The samples provide precious DNA from patients who are not necessarily eligible for surgical resection. Cytological analyses are usually performed in parallel. Blood samples (plasma and serum) are commonly used in clinical research as potential sources of minimally invasive specimen acquisition for DNA methylation studies. They display a high uniformity of specimen collection and preparation in comparison to any other clinical samples. However, aberrant DNA methylation may originate in any organ. It is currently difficult to envisage how a blood positive screening assay would point the clinician toward the site of malignancy. While plasma and serum samples are used to target cell-free circulating DNA from solid tumors, white blood cells are particularly appropriate for leukemia. DNA for detection of methylation abnormalities can also be extracted from other sources of biological fluids. Indeed, DNA from detached tumor cells, and free floating DNA from dead tumor cells can be retrieved in small proportion in biological fluids some of which contain aberrantly methylated DNA from diverse tumor origin [66]. For instance, saliva rinses that contain cells from oral mucosa is employed for methylation studies to assist in early oral tumor diagnosis [67]. Urine contains cancer cells from the bladder, kidney and prostate origin. Sputum and bronchial washings are often used to improve the diagnosis of lung cancer [68]. Pancreatic juice obtained by ultrasound echo-endoscopy has shown its potential use of methylated DNA markers for the diagnosis of pancreatic cancer *vs.* chronic pancreatitis [69,70].

## 3. Altered DNA Methylation, Marks for Cancer Diagnosis

Challenges in the field of biomarkers for cancer diagnosis follow a process of validation in which two unconditional criteria are evaluated to establish the potential usefulness of a biomarker: sensitivity and specificity [71]. Sensitivity is defined as the proportion of confirmed disease subjects who show positive detection of the marker whereas specificity refers to the proportion of patients, negative for the disease and tested negative for the biomarker. The ideal biomarker would display 100% of sensitivity and 100% of specificity. This would mean that no cancer patient would be negative for the test, and that no cancer free patient would be positive for this biomarker. As this biomarker does not exist yet, researchers face three choices:

- To improve existing tests using an existing biomarker;
- To discover new biomarkers with high sensitivity and specificity;
- To associate several biomarkers to compensate poor performance.

Over the last two decades the potential use of DNA methylation marks as biomarkers for cancer diagnosis has been assessed. Although much remains to be done in their validation and assessment of specificity and sensitivity, many alterations have arisen as potential markers. This section gives a rapid overview of the most common alterations in DNA methylation in cancer and their potential as diagnostic biomarkers. Our aim is not to give an exhaustive list of epigenetically altered genes but rather illustrate the comprehensive impact of such alterations on different functional groups of genes.

### 3.1. Cyclin Dependent Kinase Inhibitor, P16 ink4a

P16ink4a is a multiple tumor suppressor involved in cell cycle regulation. It inhibits the formation of an E2F–DB active transcriptional complex and promotes the formation of the Rb–E2F repressive transcriptional complex. This results in the prevention E2F-dependent transcription and blocks cell cycle progression past the G_1_/S restriction point [72]. *P16ink4a* is found hypermethylated in numerous types of tumors including 27% of colorectal cancer with a sensitivity of 70%, and a specificity of 100% from patient serum in liver cancer with a sensitivity of 73%, and in 24% of lung cancer [72–75].

### 3.2. O6-Methylguanine-DNA-Methyltransferase, MGMT

MGMT protein contributes to genome repair DNA by damage reversal. DNA repair occurs as a one-step reaction that leads to the transfer methyl or chloro-ethyl group to the active centre of the MGMT molecule. This results in restoration of guanine in the DNA and irreversible inactivation of MGMT. Therefore, MGMT is often referred to as a “suicide enzyme” [76]. This protein plays a dual role in cancer: loss of expression enhances DNA damage while increased expression enhances the risk of cancer. Many cancers display high expression levels of MGMT responsible for chemo-resistance. Brain tumors are the most documented, with an epigenetic repression of approximately 40% of patients [77]. A similar observation is reported in 46% of colorectal tumor samples [78]. Besides its potential as a diagnostic, it is shown that methylation of the *MGMT* promoter is associated with responsiveness to carmustine; and with an increase in overall survival and progression of disease [77].

### 3.3. Glutathione *S*-Transferase Pi 1, GSTP1

GSTP1 participates to cell detoxification. GSTP1 eliminates exogenous compounds by the conjugation of glutathione [79]. Numerous studies report altered expression of GSTP1 in cancers, and it is implicated in resistance to chemotherapy [80–82]. Loss of GSTP1 expression by promoter hypermethylation is a major event in prostate cancer in which *GSTP1* is found hypermethylated in 73% of cases with a sensitivity of 73%, a specificity of 100%, a positive predictive value (PPV) of 100% and a negative predictive value (NPV) of 78% [83]. *GSTP1* hypermethylation is also reported in breast carcinogenesis and large B cell lymphoma [80,84,85].

### 3.4. MutL Homolog 1, MLH1

The *MLH1* gene encodes a protein involved in the DNA mismatch repair machinery. Insertion or deletion events and base mismatches result from DNA polymerase replication errors, recognized and corrected by the DNA mismatch repair pathway (MMR). This pathway consists of 3 major heterodimeric complexes, MutL homologue (MutL)α, MutS homologue (MutS)α, and MutSβ. MLH1 is part of the MutLα complex and is responsible for the recruitment of the excision and repair machinery to the site of a non-complementary base marked by either MutSα or MutSβ [86,87]. The genomic region encoding *MLH1* is frequently hypermethylated in colon cancer with a high association with microsatellite instability (86% of cases) [88]. The aberrant DNA methylation of this region is also found at lower frequency in endometrial cancer (37.5% of primary tumors and 5.6% of metastatic lesions investigated) and in ovarian cancer (8% of patients) [89–91].

### 3.5. Breast Cancer Type 1 Susceptibility Protein, BRCA1

BRCA1 is involved in DNA double-strand break repair by sensing and signaling DNA breaks; through its interaction with numerous co-factors BRAC1 participates in double-strand break repair [92]. BRCA1 rapidly localizes at breakage sites marked with histone H2A-X and interacts with enzymes that alter chromatin and DNA structure, making surrounding DNA more accessible to repair machinery. The silencing of the *BRCA1* gene by promoter DNA hypermethylation occurs in breast cancer (13%), but depending on cancer subtypes, *BRCA1* promoter hypermethylation is present in 55% of sporadic mucinous breast carcinomas and in 67% of medullary breast carcinomas [91,93,94]. In ovarian cancer, *BRCA1* promoter is hypermethylated in 31% of sporadic ovarian carcinomas with loss of heterozygosity at *BRCA1* locus [93].

### 3.6. Septin 9, SEPT9

Septins are GTP binding proteins involved in numerous cellular functions such as cytokinesis and vesicle trafficking, as well as in microtubule and actin dynamics. Despite the fact that the exact role of septins is still a matter of intensive investigation, their relationship with cancer is well established. *SEPT9* hypermethylation is found in colorectal and head and neck cancer patients [95,96]. Moreover, carcinogenesis is associated with a change in SEPT9 isoform expression which can be explained by the methylation of an alternative promoter in breast cancer [97]. This illustrates the caution required when studying DNA methylation patterns, which can differ from one region of a gene to another. Finally, a plasma-based SEPT9 methylation-screening test displays a sensitivity of 72%, and a 90% specificity in the detection of colorectal cancer patients [98].

### 3.7. MicroRNA Encoding Genes

MicroRNAs are small non-coding RNA that target messenger RNA (mRNA) and inhibit their translation into proteins. Since a single microRNA can target numerous mRNAs, alterations in their expression during carcinogenesis is a major event, as they can affect a broad range of cellular functions. Lu *et al.* demonstrates that the expression profile of microRNAs can classify human cancer. Interestingly, this study reports that most of microRNAs have low expression levels in tumors compared to normal tissues [99]. High-throughput analyses of global microRNAs expression profiles are promising tools for cancer diagnosis as they quantify several hundreds of potential markers [99,100]. Moreover, considering the impact of microRNAs on cell regulation, the mechanisms responsible of their down-regulation are crucial in comprehending carcinogenesis. In addition to a global impairment of the maturation machinery of microRNAs, DNA methylation participates in the silencing of such molecules [101]. Saito *et al.* was the first to detail the relationship between miR-127 repression and DNA hypermethylation [102]. Other groups describe this silencing by DNA of miR-9-1, miR-124a3, miR-148a, miR-152, and miR-663 in 34%–86% of cases of a breast cancer collection [103]. Similarly, Wong *et al.* shows that the miR-34a promoter is hypermethylated in 18% of multiple myeloma [104]. We also show that miR-148a genomic sequence is hypermethylated in pancreatic cancer cells [35,105]. This illustrates that similarly to protein encoding regions, the silencing of non-coding RNA can be an indicator of cancer development, therefore unveiling miRNAs as remarkable source as diagnostic markers.

### 3.8. Hypomethylated Genes in Cancer

DNA demethylation associated with cancer occurs principally in long repetitive elements, and peri-centromeric regions [18]. For a long time, marker discovery process was dependent of the observation of a change in protein expression and the research of the mechanism involved. As DNA hypomethylated regions in cancer do not always correspond to protein encoding regions, their interest has been underestimated. S100 calcium-binding protein P (S100p) over-expression in pancreatic and prostate cancers occur by DNA hypomethylation [106,107]. S100p stimulates cell proliferation and survival. Its hypomethylation is found in 100% of a small cohort of pancreatic cancer tissues [106]. A lower frequency is found in prostate cancer, in which S100p is retrieved hypomethylated in 50% of samples [107]. Long Interspaced Nucleotide Elements (LINE-1) is an ancestral repetitive element containing a high concentration of CpG islands, for which the methylation level is reported as a good indicator of the global methylation level in the genome. In their study, Ogino *et al.* reports that LINE-1 hypomethylation is associated with shorter survival among colon cancer patients [108].

### 3.9. Imprinted Genes

In contrast to bi-allelic expressed genes, imprinted genes show a parental-specific mono-allelic expression, as one other allele is repressed by DNA methylation. One interesting feature of gene imprinting is that repression is not gene-specific, but depends on the imprint on the surrounding genomic region. Until now, ~130 imprinted genes have been described, many of which control crucial functions during embryonic development [109]. As carcinogenesis globally impacts the DNA methylome, one could hypothesize a probable deregulation in genomic imprinting. It has been reported that a “loss of imprinting” of insulin-like growth factor II (IGF2) and the *H19* large intergenic non-coding RNA coding regions is found in 100% of chronic myeloid leukemia (CML), 80% of ovarian tumors, 70% of Wilms’ tumors, 66% of colorectal cancer, 56% of Barrett’s esophagus, 50% of renal-cell carcinomas, 50% of esophageal cancer, 47%–85% of lung adenocarcinoma and 30% of meningioma [110]. In accordance with other hypomethylated genes in cancer, loss of imprinting impacts numerous types of cancer and represents a potential mark for further investigation.

## 4. DNA Methylation as Biomarkers

Since sensitivity and resolution in the approaches to study DNA methylation were greatly improved, a profusion of methylation marks in most types of cancer have been described in the literature [91,111]. Frequently documented DNA methylation marks are often not specific of one cancer but mostly conserved among tumor types. Thus, it seems challenging to propose a single DNA methylation alteration as a biomarker for a certain type of cancer. A combination of methylation marks is more likely to discriminate various types of cancers and to compensate the lack of specificity of each mark taken independently. Meanwhile, the combination of different biomarkers leads to a better specificity but also to lower sensitivity.

### 4.1. In Cancer Diagnosis

**Figure 1:**
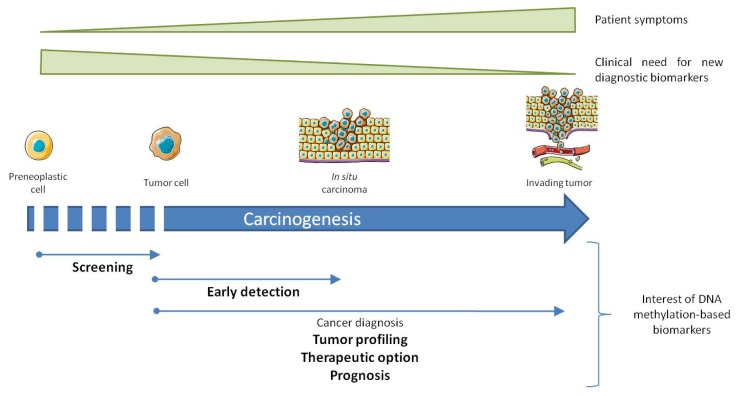
Interest of DNA methylation biomarker in cancer diagnosis. Thin arrows illustrate distinctive phases of carcinogenesis for which DNA methylation conveys improvement or an additional value to cancer diagnosis. The relative importance of DNA methylation based biomarkers regarding clinical need is bolded (high interest) or not (modest interest).

As diagnostic tools exist for the vast majority of cancers, DNA methylation-based biomarkers have to overcome current limitations and meet a clear medical need for their approval (Figure 1). They should exhibit a greater sensitivity and specificity than existing diagnostic procedure or a better accessibility through minimal invasive approaches. Thus, the actual interest of DNA methylation marker is discrete concerning cancer for which reliable diagnostic markers already exist. However, DNA methylation markers are of great interest in cases where differential diagnosis is difficult using conventional diagnostic procedure. They can be used alone or in combination with other diagnostic methods. An illustration for the use of a DNA methylation marker as a tool to differentiate cancer diagnosis, McCluskey *et al.* shows that the difference in *p16* gene hypermethylation distinguishes benign and malignant ovarian tumors and that the distal promoter is methylated in 33% of low-malignant potential tumors compared to 5% of carcinomas [112]. Similarly, our group demonstrates that the level of methylation in the gene encoding the miR-148a is a potential diagnostic tool for the differential diagnosis between pancreatic cancer and chronic pancreatitis [35].

### 4.2. In Early Detection of Cancer and Screening of High Risk Population

The early detection of cancer in the absence of specific symptoms, or screening large population cohorts represents a confounding issue in the field of cancer diagnosis (Figure 1). Both encounter the same issues regarding specificity and sensitivity as cancer diagnostic tools, but present more prerequisites. Besides the fact that alterations have to occur early during carcinogenesis, a vast proportion of patients will obviously be negative for the screening, so the test performed will have to respect a high cost/effectiveness ratio, and will rely on non-invasively obtained tissues. In population screening procedures, biomarkers with a high sensitivity will be required to discard the false positive patients. Several works have demonstrated the feasibility of cancer detection by DNA methylation detection using minimally invasive procedures. Fujiwara *et al.* determined the DNA methylation profile of 5 genes, suitable for early detection of lung cancer patients. The study shows that when DNA hypermethylation of at least one gene is considered as positive, specificity and predictive values of methylation are 85% and 75%, respectively [113]. Likewise, Müller *et al.* reports that *SFRP2* is hypermethylated in fecal DNA of patients with colorectal cancer with a sensitivity of 77% and a specificity of 77% [114]. Both studies demonstrate the feasibility of cancer diagnosis from easy-to-access samples with comfortable specificity and sensitivity, at least from a technological point of view.

### 4.3. As an Advanced Diagnostic Tool

At the frontier of cancer diagnosis, tumor profiling gives determinant information about the aggressiveness, chemo-sensitivity and invasion capacity (Figure 1). Similarly to cancer diagnosis, tumor sub-classification requires additional biomarkers (cytological, molecular) to determine the precise cellular origin and the mutational status of the tumor. DNA methylation patterns can serve as a powerful tool for improved classification of tumors. For instance, the use of high resolution DNA methylation based biomarkers (67,487 probes) in 49 acute lymphoblastic leukemia (ALL) patients can separate the different ALL subtypes and influence clinical outcome [115]. AML is a heterogeneous disease, displaying variability in the degree of commitment and differentiation of the cell lineage, representing a critical issue toward the development of accurate clinical classification, risk stratification, and targeted therapy. Figueroa *et al.* classifies 344 patients into 16 groups based on the epigenetic profiles of 15 genes. Among these groups, five display an original methylation signature with distinct clinical outcomes [116].

Similarly to AML, gliomas are a heterogeneous group of intracranial neoplasia of glial origin that can be divided into different subtypes based on their cellular origin, and, into different grades according to cell density, nuclear appearances, pleomorphism, mitotic activity, vascular proliferation and regional necrosis. Genome wide-DNA methylation studies have permitted the identification of CpG island methylator phenotype (CIMP) [117]. Uhlmann *et al.* established tissue as well as grade specific methylation profiles by the determination of the methylation level of 15 genes [118]. Another example is the identification by genome wide DNA methylation approaches of two different CIMP groups (high and low) in colorectal patients [119].

Beside tumor classification and identification, an interesting feature of DNA methylation profiles is the capacity to predict tumor response to treatments and to improve patient prognoses. Predictive epigenetic biomarkers will allow a personalized management of the patient based on their individual methylation profile. For instance, DNA hypermethylation of *MGMT* promoter in 40% of glioma patients is directly associated with the tumor resistance to conventional chemotherapy based on alkylating agents. Moreover, accumulation of normal cells in the tumor biases the assessment of MGMT expression in the tumor. *MGMT* methylation status seems to be a better indicator of its transcriptional activity. More importantly, it correlates with tumor regression and prolonged overall and disease-free survival [77].

Complexity and abundance of methylation marks revealed by genome-wide analysis open a new avenue of research towards the identification of novel biomarkers for cancer diagnosis. One could expect that the use of these approaches will lead to a “one-step diagnostic tool” informative on tumor presence, origin, sub-classification, grade, invasiveness, metastatic potential, chemo-sensitivity and relapse risk.

## 5. Principal Requirements to Develop Diagnostic Biomarkers

Despite the plethora of studies on potential methylated DNA markers for cancer diagnosis, the majority fails to meet clinical requirements. Many studies suffer defects in their design or their restricted size in patient sets; while most studies are abandoned in early validation steps. Here, we briefly describe some principal prerequisites for the elaboration of a reliable cancer diagnostic biomarker for clinical use.

### 5.1. Preclinical Requirements

The choice of tissue origin is determinant for further clinical use as early diagnosis or diagnostic biomarkers. The accessibility to biological samples is then a determinant criterion. Most studies that identify epigenetic biomarkers are conducted on resected primary tumors. These samples will not necessarily be suitable for early diagnosis in clinical routine since most tumor diagnoses are established from biopsies. Furthermore, epigenetic marks determined in the resected tissue will not always be present in easy-to-access body fluids. The less invasive the test is, the more suitable it is for clinical purpose. Thus, researchers should favor biomarker identification studies from relatively easy-to-access biological samples to permit an easier translation towards clinical use.

The very first step of biomarker discovery is often a comparison between cancer and normal tissues. A vast majority of studies present results obtained from a limited number of control tissues that may not be representative of the actual DNA methylation status in the healthy population. The concept of healthy population raises another problem: the nature of the control tissue itself. As DNA methylation patterns vary depending on age, gender, or different non-cancerous pathologies, one should be attentive to the proper definition of the control population chosen to be compared to the cancer population. Cancer free samples should be sex - and age- matched and all the essential epidemiological information should be available for the investigated cancer type to allow for proper adjustments. Researchers should ensure that DNA methylation pattern of a specific gene is different from a control free cancer population or related associated pathology. For solid tumors, a frequently used procedure to identify distinctive epigenetic marks is to compare resected tumor samples to adjacent “healthy” tissue. Unfortunately, patients that undergo surgery may harbor other alterations in resection margins due to inflammation, “driver” or “passenger” molecular alterations preceding cytological changes. The lack of control samples from healthy donors is a major limitation in biomarker identification. In this context, studies conducted from serum or samples obtained from minimally invasive procedure should abrogate this problem as they give an easier access to samples from healthy donors.

### 5.2. Validation

The main barrier in biomarker validation is certainly the reproducibility of results. This validation must follow basics rules [120]. Among all the different biases encountered during biomarker validation, over-fitting is one of the most substantial. Over-fitting occurs when a large number of variables are used to discriminate between small numbers of patients. Classically, a distinctive pattern might be independent of cancer itself, but representative of training set heterogeneity. This situation harmonizes with the use of high throughput technologies as microarrays or NGS. Then, intra-laboratory use of a large validation set, independent from the training set, is critical for the validation of the lead.

Many phase I pre-clinical studies (discovery, or proof-of-concept phase) propose that their lead can represent a new biomarker of interest for the diagnosis of a particular cancer. However, most of them do not compare their newly-identified biomarker to pre-existing diagnostic methods. The Food and Drug Administration (FDA) and other regulatory administrations request a lead to rejoin an unmet medical need, as a *sine qua non* condition in order to not be redundant with an existing standardized method. Assessment of this criterion inevitably goes through the comparison of the lead with the gold standard method clinically used where lead has the obligation to bring an improvement such as performance (sensitivity/specificity), cost/effectiveness ratio, invasiveness. Once the interest of the lead is established, results have to succeed an inter-laboratory result validation using a different cohort. Lack in this validation procedure or insufficient performance can explain why most newly-identified epigenetic biomarkers do not cross the clinical barrier.

### 5.3. Clinical Requirements

The next step of clinical implementation for a new biomarker is the extension of the validation process described above to a larger cohort to further assess inter-individual variability in DNA methylation levels. This critical stage requires a standardization of DNA methylation assessment as that extension may be multi-centered. As discussed in the “Detection of Aberrantly Methylated DNA in Biological Samples” section, reproducibility of epigenetic biomarker detection should standardize specimen sampling, reception, storage and preparation. For example, as a DNA methylation pattern is often heterogeneous among the same gene promoter, variations in technological approaches or location of analyzed promoter regions may generate different results. To date, several ready-to-use kits are available to analyze the DNA methylation status of specific genes for cancer diagnosis in clinical use [121]. Such standardization in the processing of samples would be the cobblestone of reproducible results among laboratories.

Last, concerning early detection biomarkers, an adequate follow-up for the cancer free samples used as the control population is necessary to ensure the absence of tumor development in the years following biomarker assessment. This consideration strengthens the robustness of the biomarker in detecting developing tumors at early step through the exclusion of false negative control patients.

## 6. Current Drugs/DNA Methylation Inhibitors and Clinical Trials, DNA Methylation Inhibitors

DNA methylation machinery is an attractive therapeutic target to reactivate aberrantly methylated TSG. DNA methylation inhibitors are classified as nucleosidic and non-nucleosidic inhibitors. Although many of them mediate inhibitory effects of DNA methylation in preclinical studies, only few of them are clinically used. We describe here the classical DNA methylation inhibitors as well as promising compounds that are, for some of them, currently tested in clinical trials.

### 6.1. Nucleosidic DNA Methylation Inhibitors

There exist three families of first generation nucleosidic inhibitors. They all require their incorporation in replicating DNA to be active via a covalent interaction with DNMTs. One of the main goals is to ideally target replicating tumor cells, avoiding normal cells. By interfering with the copying of aberrant DNA methylation patterns, nucleosidic inhibitors are aimed at erasing aberrant DNA hypermethylation.

#### 6.1.1. Azacytidine

Azacytidine or 5-azacytidine (Vidaza^®^, Celgene, Summit, NJ, USA) is a cytidine analog in which the carbon atom 5 is replaced by a nitrogen atom. After cell entry, azacytidine is converted into a tri-phosphorylated active form and then incorporated into DNA and RNA [122]. This analog is recognized by the DNMTs as normal cytosine, unfortunately it creates an irreversible covalent link with the enzyme leading to a cellular DNMT depletion [123,124]. Approved by the FDA, azacytidine is currently used for the treatment of AML and myelodysplastic syndrome (MDS) [125]. This compound is unstable in aqueous solution and displays significant cytotoxic effects *in vitro* and *in vitro* [123,126]. Clinical trials to test azacytidine effects on patients with relapsed or refractory myeloid malignancies (MM) are currently ongoing (ClinicalTrials.gov Identifier NCT00412919). Recent studies in xenografted mouse models demonstrate that low doses of azacytidine (and also decitabine, see below) have antitumor effects on solid tumors (breast, colon, lung) [127]. Effects of low doses of azacytidine in combination with Entinostat (HDAC inhibitor) have proven efficacy in patients with refractory advanced non-small cell lung cancer in a phase I/II study [128]. A randomized phase II clinical trial for adjuvant combined epigenetic therapy with azacytidine and Entinostat (orally bioavailabile histone deacetylase) in resected stage I non-small cell lung cancer (NCT01207726) is currently on-going. Similarly, a phase I/II clinical study of azacytidine, docetaxel and prednisone treatment of patients with metastatic prostate cancer previously treated with docetaxel is also being performed (NCT00503984). A prospective phase II study shows the safety and the efficacy of 5-days of azacytidine treatment in patients with low-risk MDS [129]. Another phase II study demonstrates the feasibility of azacytidine treatment for AML in elderly or frail patients [130]. A recently published Phase III study reports azacytidine benefit on overall survival of patients with higher-risk MDS (NCT00071799) [131].

#### 6.1.2. Decitabine

Decitabine (Dacogen^®^, MGI Pharma, Bloomington, MN, USA) or 5-aza-2′-deoxycytidine is a desoxyribose analog of cytosine. Conversely to azacytidine, which is incorporated in DNA and RNA, decitabine is only incorporated in DNA. This analog is also tri-phosphorylated to be active. It leads to DNMT depletion and genome hypomethylation. Toxic at high doses, decitabine is well tolerated at lower doses [132]. Recently, a study shows lower toxicity of a derivative of decitabine, the 2′-deoxy-5,6-dihydro-5-azacytidine at doses that induce similar DNA hypomethylation and gene reactivation [133]. Like azacytidine, decitabine is currently used for the treatment of AML and MDS [126]. Several clinical trials are now testing the effects of decitabine in combination with other drugs on solid tumors. Effects of decitabine and Peg-interferon are being evaluated on patients with melanoma (NCT00791271). Decitabine in combination with temozolomide and panobinostat is being tested for the treatment of resistant metastatic melanoma (NCT00925132). A phase I/II trial is aimed at measuring the effects of tamoxifen following epigenetic regeneration of estrogen receptor using decitabine and LBH 589 in patients with triple negative metastatic breast cancer (NCT01194908). Other analogs, such as 5-fluoro-2′-deoxycytidine, have been synthesized and are being evaluated in combination with tetrahydrouridine for head and neck neoplasm, lung neoplasm, urinary bladder and breast neoplasms (NCT00978250). Second generation analogs are also emerging. For instance, SGI-110 (Astex Pharmaceuticals), a dinucleotide “decitabine-p-deoxyguanosine” acts as a pro-drug of decitabine. It is described as an effective DNA methylation inhibitor *in vivo*, retarding tumor growth [134]. It is now being tested for the treatment of AML and MDS (NCT01261312).

#### 6.1.3. Zebularine

Zebularine or 1-(b-D-ribofuranosyl)-1,2 dihydropyrimidin-2-one (Tocris Bioscience) is a nucleoside analog of cytidine. It is a transition state analog inhibitor of cytidine deaminase (CDA) by binding to its active site [135]. Besides CDA inhibitory effects, zebularine has been demonstrated to be a DNMT inhibitor that displays antitumor activity and little toxicity [136]. Zebularine is mostly studied for its therapeutic activity on AML [137]. Preclinical studies on a Apc^(min+)^ mouse model show that long-term oral administration of zebularine causes a gender-specific abrogation of intestinal tumors while causing a tissue-specific DNA demethylation [138]. A more recent study demonstrates that in the Kasumi-1 AML cells *in vitro* model, zebularine treatment leads to different gene profiles and no hypomethylation capacity when compared to decitabine and azacytidine [139]. This study demonstrates that while they are known as DNA methylation inhibitors, the effects of these drugs are mediated by different mechanisms that probably overlap. Despite its promising tumor effects, to our knowledge, there are no clinical trials using zebularine.

These three classes of first generation nucleosides show excellent results for the treatment of AML and MDS. However, it seems important to keep in mind that these inhibitors can also lead to the demethylation and re-expression of pro-metastatic genes [140]. A need for more specific DNMT inhibitors and proper utilization of these drugs is required. Several second-generation compounds have been developed, e.g., NPEOC-DAC, CP-4200, RX-3117, thio-cytidine derivatives, *etc.*; however, despite promising preclinical results, no clinical trials have yet been initiated [141].

### 6.2. Non-Nucleoside DNA Methylation Inhibitors

Unlike nucleosidic inhibitors, the mechanism of action of non-nucleosidic DNA methylation inhibitors does not imply their incorporation into DNA molecules. For some of them the actual mechanism that leads to DNA demethylation is unclear.

#### 6.2.1. Hydralazine

Hydralazine belongs to the hydrazinophthalazine class of drug. It functions as a smooth muscle relaxant. In 1988, Cornacchia *et al.* reports that hydralazine, a drug associated with a lupus-like autoimmune disease, inhibits DNA methylation and induces self-reactivity in cloned T cell lines [142]. A later study reveals that treatment with hydralazine reactivates methylated TSG such as p16ink4a in several cell lines [143]. A phase I study shows that hydralazine treatment of four patients with cervical cancer restores the expression of methylated TSG without affecting global DNA methylation [144]. However, the exact mechanism of the DNA demethylating effects of hydralazine is still not understood. A comparative study of non-nucleoside DNA methylation inhibitors even report an absence of effects on global and TSG demethylation [145]. Often used for the treatment of hypertension, the anti-tumor activity of hydralazine in combination with valproate acid is being tested in several clinical trials. For instance, a phase II trial is testing the effects of hydralazine and magnesium valproate treatment of patients with refractory solid tumors (NCT00404508). In other reported cases, the effects of hydralazine/valproate acid are evaluated in addition to conventional chemotherapies (NCT00404326).

#### 6.2.2. Procainamide Derivatives

Procaine is a well known local anesthetic that belongs to the amino ester group. Procainamide, a derivative of procaine, is commonly used for both supraventricular and ventricular arrhythmias [146]. These two drugs are demonstrated to interact with CpG rich DNA regions and lead to DNA demethylation of TSG such as the *RAR beta 2* gene [147]. Procainamide is also a specific inhibitor of DNMT1 [148]. It displays *in vitro* growth-inhibitory effects on MCF-7 cells. However, these results are contrary to another study demonstrating that procaine and its derivatives does not induce global DNA demethylation in several cell lines [149]. Recently, six conjugates of procainamide were synthesized and showed potent inhibitory effects on the DNMT3A/3L complex and DNMT1 [150]. Another procainamide derivative, IM25 was identified from a large screening effort. It exhibits high potency in GSTp1 DNA demethylation in the MCF-7 breast cancer cell model [151]. Despite these promising results, the compounds are not yet tested for their anti-tumor effects in clinical trials.

#### 6.2.3. Flavonoids

Flavonoids form a wide family of plant secondary metabolites. They are the most important plant pigments for flower coloration. The most studied flavonoids in cancer are the (−)epigallocatechin-3-*O*-gallate (EGCG) and genistein, components of green tea and soybean, respectively. A first study reveals their DNA methylation inhibitory effects on TSGs in esophageal squamous carcinoma cells [152]. Although EGCG is described as a direct inhibitor of DNMTs, the exact mechanism of action and DNA methylation inhibitory effects are still subject to controversy [145,149,153,154]. Nevertheless, several clinical trials are currently testing flavonoids as potential anti-tumor therapy. For instance, a phase II study is evaluating the benefit of a genistein treatment in patients with prostate cancer a few months prior to radical prostatectomy (NCT01126879).

#### 6.2.4. Other Inhibitors

Several other compounds like curcumin and derivatives were reported as potential DNA methylation inhibitors. Others were synthesized RG108 (phthalimido-*l*-tryptophan), MG98 (DNMT1 antisense oligonucleotide), and SGI-1027 (lipophilic quinoline) [122]. MG98 toxicity was evaluated in several phase I clinical trials in patients with AML, MDS or advanced solid tumors [155,156]. However, none of these inhibitors have entered clinical trials for anti-tumor therapy.

## 7. Conclusions

The accumulated interest for DNA methylation-based biomarkers for cancer diagnosis in the last two decades has been highly impressive. Discoveries of original DNA methylation marks follow the continuous technological improvements of DNA methylation studies, which can provide tremendous amount of data. We illustrate that most of the DNA methylation marks described in the literature are common among cancers and that few overcome the principal requirements for clinical contribution. Hence, despite the growing interest for DNA methylation biomarkers, one should stay attentive to basic clinical requirements to ensure their reliability. Accordingly, over fitting, the use of large independent cohorts, and standardization in DNA methylation level assessment have to be accounted for to propose *bona fide* biomarkers suitable for clinical cancer diagnosis.

## References

[1] Bird A. (2002). DNA methylation patterns and epigenetic memory. Genes Dev.

[2] Gopalakrishnan S., van Emburgh B.O., Robertson K.D. (2008). DNA methylation in development and human disease. Mutat. Res.

[3] Jones P.A. (2012). Functions of DNA methylation: Islands, start sites, gene bodies and beyond. Nat. Rev. Genet.

[4] Chen T., Li E. (2004). Structure and function of eukaryotic DNA methyltransferases. Curr. Top. Dev. Biol.

[5] Kinney S.R.M., Pradhan S. (2011). Regulation of expression and activity of DNA (cytosine-5) methyltransferases in mammalian cells. Prog. Mol. Biol. Transl. Sci.

[6] Bachman K.E., Rountree M.R., Baylin S.B. (2001). Dnmt3a and Dnmt3b are transcriptional repressors that exhibit unique localization properties to heterochromatin. J. Biol. Chem.

[7] Jeong S., Liang G., Sharma S., Lin J.C., Choi S.H., Han H., Yoo C.B., Egger G., Yang A.S., Jones P.A. (2009). Selective anchoring of DNA methyltransferases 3A and 3B to nucleosomes containing methylated DNA. Mol. Cell. Biol.

[8] Sharma S., de Carvalho D.D., Jeong S., Jones P.A., Liang G. (2011). Nucleosomes containing methylated DNA stabilize DNA methyltransferases 3A/3B and ensure faithful epigenetic inheritance. PLoS Genet.

[9] Okano M., Bell D.W., Haber D.A., Li E. (1999). DNA methyltransferases Dnmt3a and Dnmt3b are essential for de novo methylation and mammalian development. Cell.

[10] Li E., Bestor T.H., Jaenisch R. (1992). Targeted mutation of the DNA methyltransferase gene results in embryonic lethality. Cell.

[11] Chedin F., Lieber M.R., Hsieh C.L. (2002). The DNA methyltransferase-like protein DNMT3L stimulates de novo methylation by Dnmt3a. Proc. Natl. Acad. Sci. USA.

[12] Okano M., Xie S., Li E. (1998). Dnmt2 is not required for de novo and maintenance methylation of viral DNA in embryonic stem cells. Nucleic Acids Res.

[13] Goll M.G., Kirpekar F., Maggert K.A., Yoder J.A., Hsieh C.L., Zhang X., Golic K.G., Jacobsen S.E., Bestor T.H. (2006). Methylation of tRNAAsp by the DNA methyltransferase homolog Dnmt2. Science.

[14] Tuorto F., Liebers R., Musch T., Schaefer M., Hofmann S., Kellner S., Frye M., Helm M., Stoecklin G., Lyko F. (2012). RNA cytosine methylation by Dnmt2 and NSun2 promotes tRNA stability and protein synthesis. Nat. Struct. Mol. Biol.

[15] Jurkowski T.P., Shanmugam R., Helm M., Jeltsch A. (2012). Mapping the tRNA binding site on the surface of human DNMT2 methyltransferase. Biochemistry.

[16] Jones P.A., Baylin S.B. (2007). The epigenomics of cancer. Cell.

[17] Berdasco M., Esteller M. (2010). Aberrant epigenetic landscape in cancer: How cellular identity goes awry. Dev. Cell.

[18] Wild L., Flanagan J.M. (2010). Genome-wide hypomethylation in cancer may be a passive consequence of transformation. Biochim. Biophys. Acta.

[19] Pastor W.A., Aravind L., Rao A. (2013). TETonic shift: Biological roles of TET proteins in DNA demethylation and transcription. Nat. Rev. Mol. Cell Biol.

[20] Hashimoto H., Liu Y., Upadhyay A.K., Chang Y., Howerton S.B., Vertino P.M., Zhang X., Cheng X. (2012). Recognition and potential mechanisms for replication and erasure of cytosine hydroxymethylation. Nucleic Acids Res.

[21] Valinluck V., Sowers L.C. (2007). Endogenous cytosine damage products alter the site selectivity of human DNA maintenance methyltransferase DNMT1. Cancer Res.

[22] He Y.-F., Li B.-Z., Li Z., Liu P., Wang Y., Tang Q., Ding J., Jia Y., Chen Z., Li L. (2011). Tet-mediated formation of 5-carboxylcytosine and its excision by TDG in mammalian DNA. Science.

[23] Song C.-X., Clark T.A., Lu X.Y., Kislyuk A., Dai Q., Turner S.W., He C., Korlach J. (2012). Sensitive and specific single-molecule sequencing of 5-hydroxymethylcytosine. Nat. Methods.

[24] Sun M., Song C.X., Huang H., Frankenberger C.A., Sankarasharma D., Gomes S., Chen P., Chen J., Chada K.K., He C. (2013). HMGA2/TET1/HOXA9 signaling pathway regulates breast cancer growth and metastasis. Proc. Natl. Acad. Sci. USA.

[25] Hsu C.-H., Peng K.L., Kang M.L., Chen Y.R., Yang Y.C., Tsai C.H., Chu C.S., Jeng Y.M., Chen Y.T., Lin F.M. (2012). TET1 suppresses cancer invasion by activating the tissue inhibitors of metalloproteinases. Cell Rep.

[26] Liu C., Liu L., Chen X., Shen J., Shan J., Xu Y., Yang Z., Wu L., Xia F., Bie P. (2013). Decrease of 5-Hydroxymethylcytosine is associated with progression of hepatocellular carcinoma through downregulation of TET1. PLoS One.

[27] Maul R.W., Gearhart P.J. (2010). AID and somatic hypermutation. Adv. Immunol.

[28] Bhutani N., Brady J.J., Damian M., Sacco A., Corbel S.Y., Blau H.M. (2010). Reprogramming towards pluripotency requires AID-dependent DNA demethylation. Nature.

[29] Métivier R., Gallais R., Tiffoche C., Le Péron C., Jurkowska R.Z., Carmouche R.P., Ibberson D., Barath P., Demay F., Reid G. (2008). Cyclical DNA methylation of a transcriptionally active promoter. Nature.

[30] Kalari S., Pfeifer G.P. (2010). Identification of driver and passenger DNA methylation in cancer by epigenomic analysis. Adv. Genet.

[31] Peng D.F., Kanai Y., Sawada M., Ushijima S., Hiraoka N., Kosuge T., Hirohashi S. (2005). Increased DNA methyltransferase 1 (DNMT1) protein expression in precancerous conditions and ductal carcinomas of the pancreas. Cancer Sci.

[32] Belinsky S.A., Nikula K.J., Baylin S.B., Issa J.P. (1996). Increased cytosine DNA-methyltransferase activity is target-cell-specific and an early event in lung cancer. Proc. Natl. Acad. Sci. USA.

[33] Lopatina N.G., Vanyushin B.F., Cronin G.M., Poirier L.A. (1998). Elevated expression and altered pattern of activity of DNA methyltransferase in liver tumors of rats fed methyl-deficient diets. Carcinogenesis.

[34] Sato N., Fukushima N., Hruban R.H., Goggins M. (2007). CpG island methylation profile of pancreatic intraepithelial neoplasia. Mod. Pathol.

[35] Hanoun N., Delpu Y., Suriawinata A.A., Bournet B., Bureau C., Selves J., Tsongalis G.J., Dufresne M., Buscail L., Cordelier P. (2010). The silencing of microRNA 148a production by DNA hypermethylation is an early event in pancreatic carcinogenesis. Clin. Chem.

[36] House M.G., Guo M., Iacobuzio-Donahue C., Herman J.G. (2003). Molecular progression of promoter methylation in intraductal papillary mucinous neoplasms (IPMN) of the pancreas. Carcinogenesis.

[37] Lee J.-H., Park S.-.J., Abraham S.C., Seo J.-.S., Nam J.-.H., Choi C., Juhng S.-.W., Rashid A., Hamilton S.R., Wu T.-.T. (2004). Frequent CpG island methylation in precursor lesions and early gastric adenocarcinomas. Oncogene.

[38] Brooks J.D., Weinstein M., Lin X., Sun Y., Pin S.S., Bova G.S., Epstein J.I., Isaacs W.B., Nelson W.G. (1998). CG island methylation changes near the GSTP1 gene in prostatic intraepithelial neoplasia. Cancer Epidemiol. Biomark. Prev.

[39] Robertson K.D., Uzvolgyi E., Liang G., Talmadge C., Sumegi J., Gonzales F.A., Jones P.A. (1999). The human DNA methyltransferases (DNMTs) 1, 3a and 3b: Coordinate mRNA expression in normal tissues and overexpression in tumors. Nucleic Acids Res.

[40] El-Deiry W.S., Nelkin B.D., Celano P., Yen R.-W.C., Falco J.P., Hamilton S.R., Baylin S.B. (1991). High expression of the DNA methyltransferase gene characterizes human neoplastic cells and progression stages of colon cancer. Proc. Natl. Acad. Sci. USA.

[41] Girault I., Tozlu S., Lidereau R., Bièche I. (2003). Expression Analysis of DNA Methyltransferases 1, 3A, and 3B in Sporadic Breast Carcinomas. Clin. Cancer Res.

[42] Mizuno S., Chijiwa T., Okamura T., Akashi K., Fukumaki Y., Niho Y., Sasaki H. (2001). Expression of DNA methyltransferases DNMT1,3A, and 3B in normal hematopoiesis and in acute and chronic myelogenous leukemia. Blood.

[43] Robertson K.D. (2001). DNA methylation, methyltransferases, and cancer. Oncogene.

[44] Gaidzik V.I., Paschka P., Späth D., Habdank M., Köhne C.-H., Germing U., von Lilienfeld-Toal M., Held G., Horst H.-A., Haase D. (2012). *TET2* Mutations in Acute Myeloid Leukemia (AML): Results from a comprehensive genetic and clinical analysis of the AML study group. J. Clin. Oncol.

[45] Simó-Riudalbas L., Melo S.A., Esteller M. (2011). DNMT3B gene amplification predicts resistance to DNA demethylating drugs. Genes Chromosomes Cancer.

[46] Lopez de Silanes I., Gorospe M., Taniguchi H., Abdelmohsen K., Srikantan S., Alaminos M., Berdasco M., Urdinguio R.G., Fraga M.F., Jacinto F.V. (2009). The RNA-binding protein HuR regulates DNA methylation through stabilization of DNMT3b mRNA. Nucleic Acids Res.

[47] Ley T.J., Ding L., Walter M.J., McLellan M.D., Lamprecht T., Larson D.E., Kandoth C., Payton J.E., Baty J., Welch J. (2010). DNMT3A mutations in acute myeloid leukemia. N. Engl. J. Med.

[48] Singer J., Stellwagen R.H., Roberts-Ems J., Riggs A.D. (1977). 5-Methylcytosine content of rat hepatoma DNA substituted with bromodeoxyuridine. J. Biol. Chem.

[49] Fraga M.F., Rodríguez R., Cañal M.J. (2000). Rapid quantification of DNA methylation by high performance capillary electrophoresis. Electrophoresis.

[50] Ramsahoye B.H. (2002). Nearest-neighbor analysis. Methods Mol. Biol.

[51] Guerrero-Preston R., Santella R.M., Blanco A., Desai M., Berdasco M., Fraga M. (2007). Global DNA hypomethylation in liver cancer cases and controls: A phase I preclinical biomarker development study. Epigenetics.

[52] Costello J.F., Smiraglia D.J., Plass C. (2002). Restriction landmark genome scanning. Methods.

[53] Frigola J., Ribas M., Risques R.-A., Peinado M.A. (2002). Methylome profiling of cancer cells by amplification of inter-methylated sites (AIMS). Nucleic Acids Res.

[54] Liang G., Chan M.F., Tomigahara Y., Tsai Y.C., Gonzales F.A., Li E., Laird P.W., Jones P.A. (2002). Cooperativity between DNA methyltransferases in the maintenance methylation of repetitive elements. Mol. Cell. Biol.

[55] Frommer M., McDonald L.E., Millar D.S., Collis C.M., Watt F., Grigg G.W., Molloy P.L., Paul C.L. (1992). A genomic sequencing protocol that yields a positive display of 5-methylcytosine residues in individual DNA strands. Proc. Natl. Acad. Sci. USA.

[56] Herman J.G., Graff J.R., Myöhänen S., Nelkin B.D., Baylin S.B. (1996). Methylation-specific PCR: A novel PCR assay for methylation status of CpG islands. Proc. Natl. Acad. Sci. USA.

[57] Yamamoto N., Nakayama T., Kajita M., Miyake T., Iwamoto T., Kim S.J., Sakai A., Ishihara H., Tamaki Y., Noguchi S. (2012). Detection of aberrant promoter methylation of GSTP1, RASSF1A, and RARβ2 in serum DNA of patients with breast cancer by a newly established one-step methylation-specific PCR assay. Breast Cancer Res. Treat.

[58] Xiong Z., Laird P.W. (1997). COBRA: A sensitive and quantitative DNA methylation assay. Nucleic Acids Res.

[59] Eads C.A., Danenberg K.D., Kawakami K., Saltz L.B., Blake C., Shibata D., Danenberg P.V., Laird P.W. (2000). MethyLight: A high-throughput assay to measure DNA methylation. Nucleic Acids Res.

[60] Tost J., Gut I.G. (2007). DNA methylation analysis by pyrosequencing. Nat. Protoc.

[61] Zhang X., Yazaki J., Sundaresan A., Cokus S., Chan S.W.-.L., Chen H., Henderson I.R., Shinn P., Pellegrini M., Jacobsen S.E. (2006). Genome-wide high-resolution mapping and functional analysis of DNA methylation in arabidopsis. Cell.

[62] Taylor K.H., Kramer R.S., Davis J.W., Guo J., Duff D.J., Xu D., Caldwell C.W., Shi H. (2007). Ultradeep bisulfite sequencing analysis of DNA methylation patterns in multiple gene promoters by 454 sequencing. Cancer Res.

[63] Kulis M., Heath S., Bibikova M., Queirós A.C., Navarro A., Clot G., Martínez-Trillos A., Castellano G., Brun-Heath I., Pinyol M. (2012). Epigenomic analysis detects widespread gene-body DNA hypomethylation in chronic lymphocytic leukemia. Nat. Genet.

[64] Bibikova M., Lin Z., Zhou L., Chudin E., Garcia E.W., Wu B., Doucet D., Thomas N.J., Wang Y., Vollmer E. (2006). High-throughput DNA methylation profiling using universal bead arrays. Genome Res.

[65] Dedeurwaerder S., Desmedt C., Calonne E., Singhal S.K., Haibe-Kains B., Defrance M., Michiels S., Volkmar M., Deplus R., Luciani J. (2011). DNA methylation profiling reveals a predominant immune component in breast cancers. EMBO Mol. Med.

[66] Liloglou T., Field J.K. (2010). Detection of DNA methylation changes in body fluids. Adv. Genet.

[67] Carvalho A.L., Henrique R., Jeronimo C., Nayak C.S., Reddy A.N., Hoque M.O., Chang S., Brait M., Jiang W.-.W., Kim M.M. (2011). Detection of promoter hypermethylation in salivary rinses as a biomarker for head and neck squamous cell carcinoma surveillance. Clin. Cancer Res..

[68] Anglim P.P., Alonzo T.A., Laird-Offringa I.A. (2008). DNA methylation-based biomarkers for early detection of non-small cell lung cancer: An update. Mol. Cancer.

[69] Yan L., McFaul C., Howes N., Leslie J., Lancaster G., Wong T., Threadgold J., Evans J., Gilmore I., Smart H. (2005). Molecular analysis to detect pancreatic ductal adenocarcinoma in high-risk groups. Gastroenterology.

[70] Matsubayashi H., Canto M., Sato N., Klein A., Abe T., Yamashita K., Yeo C.J., Kalloo A., Hruban R., Goggins M. (2006). DNA methylation alterations in the pancreatic juice of patients with suspected pancreatic disease. Cancer Res.

[71] Wagner P.D., Verma M., Srivastava S. (2004). Challenges for biomarkers in cancer detection. Ann. N. Y. Acad. Sci.

[72] Rocco J.W., Sidransky D. (2001). p16(MTS-1/CDKN2/INK4a) in cancer progression. Exp. Cell Res.

[73] Zou H.-Z., Yu B.-M., Wang Z.-W., Sun J.-Y., Cang H., Gao F., Li D.H., Zhao R., Feng G.-G., Yi J. (2002). Detection of Aberrant p16 Methylation in the Serum of Colorectal Cancer Patients. Clin. Cancer Res.

[74] Wong I.H.N., Lo Y.M.D., Zhang J., Liew C.-T., Ng M.H.L., Wong N., Lai P.B.S., Lau W.Y., Hjelm N.M., Johnson P.J. (1999). Detection of Aberrant p16 Methylation in the Plasma and Serum of Liver Cancer Patients. Cancer Res.

[75] Belinsky S.A., Nikula K.J., Palmisano W.A., Michels R., Saccomanno G., Gabrielson E., Baylin S.B., Herman J.G. (1998). Aberrant methylation of p16INK4a is an early event in lung cancer and a potential biomarker for early diagnosis. Proc. Natl. Acad. Sci. USA.

[76] Kaina B., Margison G.P., Christmann M., Targeting O. (2010). 6-methylguanine-DNA methyltransferase with specific inhibitors as a strategy in cancer therapy. Cell. Mol. Life Sci.

[77] Esteller M., Garcia-Foncillas J., Andion E., Goodman S.N., Hidalgo O.F., Vanaclocha V., Baylin S.B., Herman J.G. (2000). Inactivation of the DNA-repair gene MGMT and the clinical response of gliomas to alkylating agents. N. Engl. J. Med.

[78] Shen L., Kondo Y., Rosner G.L., Xiao L., Hernandez N.S., Vilaythong J., Houlihan P.S., Krouse R.S., Prasad A.R., Einspahr J.G. (2005). MGMT promoter methylation and field defect in sporadic colorectal cancer. J. Natl. Cancer Inst..

[79] Townsend D.M., Tew K.D. (2003). The role of glutathione-S-transferase in anti-cancer drug resistance. Oncogene.

[80] Nakamichi I., Tomita Y., Zhang B., Sugiyama H., Kanakura Y., Fukuhara S., Hino M., Kanamaru A., Ogawa H., Aozasa K. (2007). Correlation between promoter hypermethylation of GSTP1 and response to chemotherapy in diffuse large B cell lymphoma. Ann. Hematol.

[81] Miyake T., Nakayama T., Naoi Y., Yamamoto N., Otani Y., Kim S.J., Shimazu K., Shimomura A., Maruyama N., Tamaki Y. (2012). GSTP1 expression predicts poor pathological complete response to neoadjuvant chemotherapy in ER-negative breast cancer. Cancer Sci..

[82] Zhang Y., Qu X., Jing W., Hu X., Yang X., Hou K., Teng Y., Zhang J., Liu Y. (2009). GSTP1 determines cis-platinum cytotoxicity in gastric adenocarcinoma MGC803 cells: Regulation by promoter methylation and extracellular regulated kinase signaling. Anticancer Drugs.

[83] Harden S.V., Guo Z., Epstein J.I., Sidransky D. (2003). Quantitative Gstp1 methylation clearly distinguishes benign prostatic tissue and limited prostate adenocarcinoma. J. Urol.

[84] Saxena A., Dhillon V.S., Shahid M., Khalil H.S., Rani M., Prasad D.A.S.T., Hedau S., Hussain A., Naqvi R.A., Deo S.V.S. (2012). GSTP1 methylation and polymorphism increase the risk of breast cancer and the effects of diet and lifestyle in breast cancer patients. Exp. Ther. Med.

[85] Hashad D.I., Hashad M.M.E.I., Talaat I.M., Ibrahim M.A. (2011). Role of glutathione-*S*-transferase P1 hypermethylation in molecular detection of prostate cancer. Genet Test Mol. Biomark.

[86] Fukui K. (2010). DNA Mismatch Repair in Eukaryotes and Bacteria. J. Nucleic Acids.

[87] Kantelinen J., Kansikas M., Korhonen M.K., Ollila S., Heinimann K., Kariola R., Nyström M. (2010). MutSbeta exceeds MutSalpha in dinucleotide loop repair. Br. J. Cancer.

[88] Menigatti M., Di Gregorio C., Borghi F., Sala E., Scarselli A., Pedroni M., Foroni M., Benatti P., Roncucci L., Ponz de Leon M. (2001). Methylation pattern of different regions of the MLH1 promoter and silencing of gene expression in hereditary and sporadic colorectal cancer. Genes Chromosomes Cancer.

[89] Bischoff J., Ignatov A., Semczuk A., Schwarzenau C., Ignatov T., Krebs T., Küster D., Przadka-Rabaniuk D., Roessner A., Costa S.D. (2012). hMLH1 promoter hypermethylation and MSI status in human endometrial carcinomas with and without metastases. Clin. Exp. Metastasis.

[90] Ozdemir F., Altinisik J., Karateke A., Coksuer H., Buyru N. (2012). Methylation of tumor suppressor genes in ovarian cancer. Exp. Ther. Med.

[91] Esteller M., Corn P.G., Baylin S.B., Herman J.G. (2001). A gene hypermethylation profile of human cancer. Cancer Res.

[92] Venkitaraman A.R. (2002). Cancer susceptibility and the functions of BRCA1 and BRCA2. Cell.

[93] Esteller M., Silva J.M., Dominguez G., Bonilla F., Matias-Guiu X., Lerma E., Bussaglia E., Prat J., Harkes I.C., Repasky E.A. (2000). Promoter Hypermethylation and BRCA1 Inactivation in Sporadic Breast and Ovarian Tumors. J. Natl. Cancer Inst..

[94] Dobrovic A., Simpfendorfer D. (1997). Methylation of the BRCA1 gene in sporadic breast cancer. Cancer Res.

[95] Lofton-Day C., Model F., DeVos T., Tetzner R., Distler J., Schuster M., Song X., Lesche R., Liebenberg V., Ebert M. (2008). DNA methylation biomarkers for blood-based colorectal cancer screening. Clin. Chem.

[96] Bennett K.L., Karpenko M., Lin M., Claus R., Arab K., Dyckhoff G., Plinkert P., Herpel E., Smiraglia D., Plass C. (2008). Frequently methylated tumor suppressor genes in head and neck squamous cell carcinoma. Cancer Res.

[97] Connolly D., Yang Z., Castaldi M., Simmons N., Oktay M.H., Coniglio S., Fazzarim M.J., Verdier-Pinard P., Montagna C. (2011). Septin 9 isoform expression, localization and epigenetic changes during human and mouse breast cancer progression. Breast Cancer Res..

[98] Grützmann R., Molnar B., Pilarsky C., Habermann J.K., Schlag P.M., Saeger H.D., Miehlke S., Stolz T., Model F., Roblick U.J. (2008). Sensitive detection of colorectal cancer in peripheral blood by septin 9 DNA methylation assay. PLoS One.

[99] Lu J., Getz G., Miska E.A., Alvarez-Saavedra E., Lamb J., Peck D., Sweet-Cordero A., Ebert B.L., Mak R.H., Ferrando A.A. (2005). MicroRNA expression profiles classify human cancers. Nature.

[100] Iorio M.V., Visone R., Leva G.D., Donati V., Petrocca F., Casalini P., Taccioli C., Volinia S., Liu C.-G., Alder H. (2007). MicroRNA signatures in human ovarian cancer. Cancer Res.

[101] Weber B., Stresemann C., Brueckner B., Lyko F. (2007). Methylation of human microRNA genes in normal and neoplastic cells. Cell Cycle.

[102] Saito Y., Liang G., Egger G., Friedman J.M., Chuang J.C., Coetzee G.A., Jones P.A. (2006). Specific activation of microRNA-127 with downregulation of the proto-oncogene BCL6 by chromatin-modifying drugs in human cancer cells. Cancer Cell.

[103] Lehmann U., Hasemeier B., Christgen M., Müller M., Römermann D., Länger F., Kreipe H. (2008). Epigenetic inactivation of microRNA gene hsa-mir-9-1 in human breast cancer. J. Pathol.

[104] Wong K.Y., Huang X., Chim C.S. (2012). DNA methylation of microRNA genes in multiple myeloma. Carcinogenesis.

[105] Cordelier P., Torrisani J., Cho W.C.S. (2011). MicroRNAs in Pancreatic Cancer: Potential Interests as Biomarkers and Therapeutic Tools. MicroRNAs in Cancer Translational Research.

[106] Sato N., Fukushima N., Matsubayashi H., Goggins M. (2004). Identification of maspin and S100P as novel hypomethylation targets in pancreatic cancer using global gene expression profiling. Oncogene.

[107] Wang Q., Williamson M., Bott S., Brookman-Amissah N., Freeman A., Nariculam J., Hubank M.J.F., Ahmed A., Masters J.R. (2007). Hypomethylation of WNT5A, CRIP1 and S100P in prostate cancer. Oncogene.

[108] Ogino S., Nosho K., Kirkner G.J., Kawasaki T., Chan A.T., Schernhammer E.S., Giovannucci E.L., Fuchs C.S. (2008). A cohort study of tumoral LINE-1 hypomethylation and prognosis in colon cancer. J. Natl. Cancer Inst.

[109] Koerner M.V., Barlow D.P. (2010). Genomic imprinting—an epigenetic gene-regulatory model. Curr. Opin. Genet. Develop.

[110] Jelinic P., Shaw P. (2007). Loss of imprinting and cancer. J. Pathol.

[111] Mulero-Navarro S., Esteller M. (2008). Epigenetic biomarkers for human cancer: The time is now. Crit. Rev. Oncol. Hematol.

[112] McCluskey L.L., Chen C., Delgadillo E., Felix J.C., Muderspach L.I., Dubeau L. (1999). Differences inp16Gene methylation and expression in benign and malignant ovarian tumors. Gynecol. Oncol.

[113] Fujiwara K., Fujimoto N., Tabata M., Nishii K., Matsuo K., Hotta K., Kozuki T., Aoe M., Kiura K., Ueoka H. (2005). Identification of epigenetic aberrant promoter methylation in serum DNA is useful for early detection of lung cancer. Clin. Cancer Res.

[114] Müller H.M., Oberwalder M., Fiegl H., Morandell M., Goebel G., Zitt M., Mühlthaler M., Öfner D., Margreiter R., Widschwendter M. (2004). Methylation changes in faecal DNA: A marker for colorectal cancer screening?. Lancet.

[115] Stumpel D.J.P.M., Schneider P., van Roon E.H.J., Boer J.M., de Lorenzo P., Valsecchi M.G., de Menezes R.X., Pieters R., Stam R.W. (2009). Specific promoter methylation identifies different subgroups of MLL-rearranged infant acute lymphoblastic leukemia, influences clinical outcome, and provides therapeutic options. Blood.

[116] Figueroa M.E., Lugthart S., Li Y., Erpelinck-Verschueren C., Deng X., Christos P.J., Schifano E., Booth J., van Putten W., Skrabanek L. (2010). DNA methylation signatures identify biologically distinct subtypes in acute myeloid leukemia. Cancer Cell.

[117] Noushmehr H., Weisenberger D.J., Diefes K., Phillips H.S., Pujara K., Berman B.P., Pan F., Pelloski C.E., Sulman E.P., Bhat K.P. (2010). Identification of a CpG island methylator phenotype that defines a distinct subgroup of glioma. Cancer Cell.

[118] Uhlmann K., Rohde K., Zeller C., Szymas J., Vogel S., Marczinek K., Thiel G., Nürnberg P., Laird P.W. (2003). Distinct methylation profiles of glioma subtypes. Int. J. Cancer.

[119] Hinoue T., Weisenberger D.J., Lange C.P.E., Shen H., Byun H.-M., Van Den Berg D., Malik S., Pan F., Noushmehr H., van Dijk C.M. (2012). Genome-scale analysis of aberrant DNA methylation in colorectal cancer. Genome Res.

[120] Ransohoff D.F. (2004). Rules of evidence for cancer molecular-marker discovery and validation. Nat. Rev. Cancer.

[121] Mikeska T., Bock C., Do H., Dobrovic A. (2012). DNA methylation biomarkers in cancer: Progress towards clinical implementation. Expert Rev. Mol. Diagn.

[122] Gros C., Fahy J., Halby L., Dufau I., Erdmann A., Gregoire J.-M., Ausseil F., Vispé S., Arimondo P.B. (2012). DNA methylation inhibitors in cancer: Recent and future approaches. Biochimie.

[123] Santi D.V., Norment A., Garrett C.E. (1984). Covalent bond formation between a DNA-cytosine methyltransferase and DNA containing 5-azacytosine. Proc. Natl. Acad. Sci. USA.

[124] Cheng J.C., Yoo C.B., Weisenberger D.J., Chuang J., Wozniak C., Liang G., Marquez V.E., Greer S., Orntoft T.F., Thykjaer T. (2004). Preferential response of cancer cells to zebularine. Cancer Cell.

[125] Lübbert M. (2000). DNA methylation inhibitors in the treatment of leukemias, myelodysplastic syndromes and hemoglobinopathies: Clinical results and possible mechanisms of action. Curr. Top. Microbiol. Immunol.

[126] Robak T. (2011). New nucleoside analogs for patients with hematological malignancies. Expert Opin. Investig. Drugs.

[127] Tsai H.-C., Li H., Van Neste L., Cai Y., Robert C., Rassool F.V., Shin J.J., Harbom K.M., Beaty R., Pappou E. (2012). Transient low doses of DNA-demethylating agents exert durable antitumor effects on hematological and epithelial tumor cells. Cancer Cell.

[128] Juergens R.A., Wrangle J., Vendetti F.P., Murphy S.C., Zhao M., Coleman B., Sebree R., Rodgers K., Hooker C.M., Franco N. (2011). Combination epigenetic therapy has efficacy in patients with refractory advanced non-small cell lung cancer. Cancer Discov.

[129] Filì C., Malagola M., Follo M.Y., Finelli C., Iacobucci I., Martinelli G., Cattina F., Clissa C., Candoni A., Fanin R. (2013). Prospective phase II study on 5-days azacitidine for treatment of symptomatic and/or erythropoietin unresponsive patients with low/INT-1–risk myelodysplastic syndromes. Clin. Cancer Res.

[130] Passweg J.R., Pabst T., Blum S., Bargetzi M., Li Q., Heim D., Stussi G., Gregor M., Leoncini L., Meyer-Monard S. (2013). Azacytidine for acute myeloid leukemia in elderly or frail patients: A phase II trial (SAKK 30/07). Leuk. Lymphoma.

[131] Gore S.D., Fenaux P., Santini V., Bennett J.M., Silverman L.R., Seymour J.F., Hellstrom-Lindberg E., Swern A.S., Beach C.L., List A.F. (2013). A multivariate analysis of the relationship between response and survival among patients with higher-risk myelodysplastic syndromes treated within azacitidine or conventional care regimens in the randomized AZA-001 trial. Haematologica.

[132] Bryan J., Kantarjian H., Garcia-Manero G., Jabbour E. (2011). Pharmacokinetic evaluation of decitabine for the treatment of leukemia. Expert Opin. Drug Metab. Toxicol.

[133] Matoušová M., Votruba I., Otmar M., Tloušt’ová E., Günterová J., Mertlíková-Kaiserová H. (2011). 2′-deoxy-5,6-dihydro-5-azacytidine—a less toxic alternative of 2′-deoxy-5-azacytidine: A comparative study of hypomethylating potential. Epigenetics.

[134] Chuang J.C., Warner S.L., Vollmer D., Vankayalapati H., Redkar S., Bearss D.J., Qiu X., Yoo C.B., Jones P.A. (2010). S110, a 5-Aza-2′-deoxycytidine-containing dinucleotide, is an effective DNA methylation inhibitor *in vivo* and can reduce tumor growth. Mol. Cancer Ther.

[135] Schroeder G.K., Zhou L., Snider M.J., Chen X., Wolfenden R. (2012). Flight of a cytidine deaminase complex with an imperfect transition state analogue inhibitor: Mass spectrometric evidence for the presence of a trapped water molecule. Biochemistry.

[136] Zhou L., Cheng X., Connolly B.A., Dickman M.J., Hurd P.J., Hornby D.P. (2002). Zebularine: A novel DNA methylation inhibitor that forms a covalent complex with DNA methyltransferases. J. Mol. Biol.

[137] Scott S.A., Lakshimikuttysamma A., Sheridan D.P., Sanche S.E., Geyer C.R., DeCoteau J.F. (2007). Zebularine inhibits human acute myeloid leukemia cell growth *in vitro* in association with p15INK4B demethylation and reexpression. Exp. Hematol.

[138] Yoo C.B., Chuang J.C., Byun H.-M., Egger G., Yang A.S., Dubeau L., Long T., Laird P.W., Marquez V.E., Jones P.A. (2008). Long-term epigenetic therapy with oral zebularine has minimal side effects and prevents intestinal tumors in mice. Cancer Prev. Res.

[139] Flotho C., Claus R., Batz C., Schneider M., Sandrock I., Ihde S., Plass C., Niemeyer C.M., Lübbert M. (2009). The DNA methyltransferase inhibitors azacitidine, decitabine and zebularine exert differential effects on cancer gene expression in acute myeloid leukemia cells. Leukemia.

[140] Chik F., Szyf M. (2011). Effects of specific DNMT gene depletion on cancer cell transformation and breast cancer cell invasion; toward selective DNMT inhibitors. Carcinogenesis.

[141] Fahy J., Jeltsch A., Arimondo P.B. (2012). DNA methyltransferase inhibitors in cancer: A chemical and therapeutic patent overview and selected clinical studies. Expert Opin. Ther. Pat.

[142] Cornacchia E., Golbus J., Maybaum J., Strahler J., Hanash S., Richardson B. (1988). Hydralazine and procainamide inhibit T cell DNA methylation and induce autoreactivity. J. Immunol.

[143] Segura-Pacheco B., Trejo-Becerril C., Perez-Cardenas E., Taja-Chayeb L., Mariscal I., Chavez A., Acuña C., Salazar A.M., Lizano M., Dueñas-Gonzalez A. (2003). Reactivation of tumor suppressor genes by the cardiovascular drugs hydralazine and procainamide and their potential use in cancer therapy. Clin. Cancer Res.

[144] Zambrano P., Segura-Pacheco B., Perez-Cardenas E., Cetina L., Revilla-Vazquez A., Taja-Chayeb L., Chavez-Blanco A., Angeles E., Cabrera G., Sandoval K. (2005). A phase I study of hydralazine to demethylate and reactivate the expression of tumor suppressor genes. BMC Cancer.

[145] Chuang J.C., Yoo C.B., Kwan J.M., Li T.W.H., Liang G., Yang A.S., Jones P.A. (2005). Comparison of biological effects of non-nucleoside DNA methylation inhibitors *versus* 5-aza-2′-deoxycytidine. Mol. Cancer Ther.

[146] Fenster P.E., Comess K.A., Marsh R., Katzenberg C., Hager W.D. (1983). Conversion of atrial fibrillation to sinus rhythm by acute intravenous procainamide infusion. Am. Heart J.

[147] Villar-Garea A., Fraga M.F., Espada J., Esteller M. (2003). Procaine is a DNA-demethylating agent with growth-inhibitory effects in human cancer cells. Cancer Res.

[148] Lee B.H., Yegnasubramanian S., Lin X., Nelson W.G. (2005). Procainamide is a specific inhibitor of DNA methyltransferase 1. J. Biol. Chem.

[149] Stresemann C., Brueckner B., Musch T., Stopper H., Lyko F. (2006). Functional diversity of DNA methyltransferase inhibitors in human cancer cell lines. Cancer Res.

[150] Halby L., Champion C., Sénamaud-Beaufort C., Ajjan S., Drujon T., Rajavelu A., Ceccaldi A., Jurkowska R., Lequin O., Nelson W.G. (2012). Rapid synthesis of new DNMT inhibitors derivatives of procainamide. Chembiochem.

[151] Lin Y.-S., Shaw A.Y., Wang S.-G., Hsu C.-C., Teng I.-W., Tseng M.-J., Huang T.H., Chen C.-S., Leu Y.-W., Hsiao S.-H. (2011). Identification of novel DNA methylation inhibitors via a two-component reporter gene system. J. Biomed. Sci.

[152] Fang M.Z., Chen D., Sun Y., Jin Z., Christman J.K., Yang C.S. (2005). Reversal of hypermethylation and reactivation of p16INK4a, RARbeta, and MGMT genes by genistein and other isoflavones from soy. Clin. Cancer Res.

[153] Lee W.J., Shim J.-Y., Zhu B.T. (2005). Mechanisms for the inhibition of DNA methyltransferases by tea catechins and bioflavonoids. Mol. Pharmacol.

[154] Wang Y., Li Y., Liu X., Cho W.C. (2013). Genetic and epigenetic studies for determining molecular targets of natural product anticancer agents. Curr. Cancer Drug Targets.

[155] Plummer R., Vidal L., Griffin M., Lesley M., de Bono J., Coulthard S., Sludden J., Siu L.L., Chen E.X., Oza A.M. (2009). Phase I study of MG98, an oligonucleotide antisense inhibitor of human DNA methyltransferase 1, given as a 7-day infusion in patients with advanced solid tumors. Clin. Cancer Res.

[156] Klisovic R.B., Stock W., Cataland S., Klisovic M.I., Liu S., Blum W., Green M., Odenike O., Godley L., Burgt J.V. (2008). A phase I biological study of MG98, an oligodeoxynucleotide antisense to DNA methyltransferase 1, in patients with high-risk myelodysplasia and acute myeloid leukemia. Clin. Cancer Res.

